# Schisandrae Fructus Reduces Symptoms of 4-Vinylcyclohexene Diepoxide-Induced Ovarian Failure in Mice

**DOI:** 10.1155/2017/2564787

**Published:** 2017-05-11

**Authors:** Dasom Shin, Jinhee Ha, Seong Bin Hong, Geun-Hyung Kang, Deok-Sang Hwang, Hyunsu Bae

**Affiliations:** ^1^Department of Science in Korean Medicine, Graduate School, Kyung Hee University, 26 Kyungheedae-ro, Dongdaemoon-gu, Seoul 02447, Republic of Korea; ^2^Biomix Inc., Goyang, Gyeonggi-do, Republic of Korea; ^3^Department of Obstetrics and Gynecology, College of Korean Medicine, Kyung Hee University, Seoul, Republic of Korea

## Abstract

Menopause is associated with a decrease in the level of sex hormones such as ovarian estradiol and progesterone and can cause various symptoms such as depression, hot flash, fatigue, heart palpitations, and headache. Furthermore, there is a risk of developing complications such as osteoporosis, cardiovascular diseases, Alzheimer's disease, and ovarian cancer. Schisandrae Fructus (SF) is widely used in Korean medicine as a cure for such complications. This study was conducted to evaluate the therapeutic effects of SF against menopause symptoms associated with follicle depletion caused by the industrial chemical 4-vinylcyclohexene diepoxide (VCD) in mice. VCD directly targets the preantral follicles. Mice were injected with VCD (160 mg/kg intraperitoneally) daily for 15 days and then with SF dosage 3 times/week for six weeks. To evaluate the effects of SF, body weight, tail skin temperature, uterine weight, lipid profile, and osteocalcin levels were measured. A decrease in body weight and tail skin temperature and an increase in uterine weight were observed upon SF treatment. Moreover, SF treatment significantly decreased total cholesterol, triglyceride, osteocalcin, and low-density lipoprotein levels and low-density/high-density lipoprotein ratio. These results suggest the potential use of SF in the treatment of menopausal symptoms in women.

## 1. Introduction

Menopause is a natural process experienced by women [1]. It is caused by cessation of estrogen production by the ovaries [2]. Symptoms include hot flashes, anxiety, depression, night sweating, insomnia, vaginal dryness, and joint pain [2, 3]. Furthermore, complications such as osteoporosis, cardiovascular disease [4], Alzheimer's disease [5], and ovarian cancer [6] may follow. Hormone replacement therapy (HRT) is employed to ameliorate menopausal symptoms in many menopausal women. However, HRT is known to cause breast cancer, ovarian cancer, uterine cancer, and gallbladder diseases [7–9].

In a previous study, an ovariectomy model (OVX) and a chemical model [4-vinylcyclohexene diepoxide (VCD)] were used to study menopause in women [10]. The OVX model is a common surgically induced animal model for studying menopause and used in the field of aging, such as mechanisms for skeletal responses to estrogen deficiency [11, 12]. In the OVX model, ovarian hormones are removed rapidly rather than the gradual decline that occurs in natural menopause and the symptom is similar to women who undergo surgical menopause [10]. A chemical model is developed for perimenopause and postmenopause by administrating the diepoxide metabolite, 4-vinylcyclohexene (VCD) [13]. VCD is industrially used as a cross linking agent for production of epoxy resins [10, 14]. VCD exposure selectively accelerates the natural loss of small primordial and primary ovarian follicles of mice without affecting other tissues [10, 15, 16]. Most of women undergo natural menopause and only less than 13% of women undergo surgical menopause [17]. Therefore, VCD-induced ovarian failure is a useful tool to mimic human menopause and retains residual ovarian tissues. We used the chemical model as it is closely related to mimic human menopausal transition [1].

Schisandrae Fructus (SF), which belongs to the Magnoliaceae family, is well known in Korean medicine. SF comprises sweet (fruit skin), sour (pulp), bitter/pungent (seed core), and salty components (all parts) [18, 19]. According to previous studies, It can also be used as antioxidant [20], antitumor [21], and antiaging agent [22] and in the treatment of liver diseases [23]. In Korea, SF has been used in various herbal prescriptions for treating menopausal symptoms [18]. The objective of this study was to analyze the effects of SF on menopausal symptoms in VCD-induced ovarian failure mice model.

## 2. Materials and Methods

### 2.1. Chemicals and Reagents

VCD (94956) and 17*β*-estradiol (E2; E8875) were purchased from Sigma-Aldrich Korea (Seoul, South Korea). VCD was dissolved in sesame oil (160 mg/kg; S3547, Sigma-Aldrich) and E2 in EtOH (0.5 mg/kg).

### 2.2. Extraction of SF

The extract of SF was provided from Biomix (Goyang, Gyeonggi-do, Republic of Korea). A decoction of the traditional medicine was prepared by extracting it with 30% EtOH for 3 h. Moreover, the remains of the herbs and impurities were separated from the extracted liquid by a filtration separation process. After filtration and drying, extracts of SF were remained liquefied. All extracting processes of it were controlled by Biomix Inc. SF was carefully measured to prepare the necessary doses for each treatment group and then dissolved in distilled water for 30 min at a controlled room temperature. SF was orally administered to mice at 25, 100, and 400 mg/kg/day for 6 weeks.

### 2.3. High-Performance Liquid Chromatographic Analysis of SF

**Figure 1:**
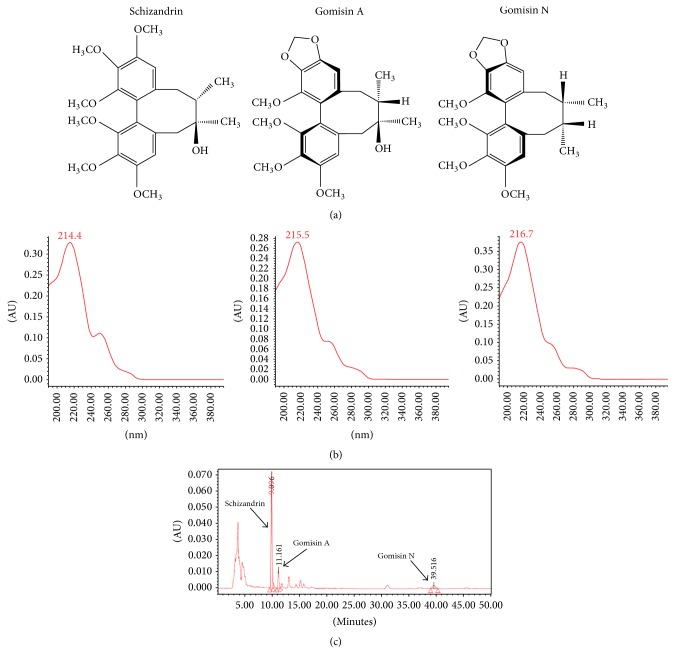
The structure of Schizandrin, Gomisin A, and Gomisin N (a), UV spectrum of Schizandrin, Gomisin A, and Gomisin N (b), and chromatogram of SF (c) using HPLC systems.

Gomisin N was purchased from the National Development Institute of Korean Medicine. HPLC-grade acetonitrile was purchased from Daejung Inc. The compound structure is presented in Figure 1. The purity of this standard compound was determined to be greater than 97% by normalization of the peak areas detected by HPLC analysis. SF was dissolved in 20 ml of methanol and sonicated for 30 min. The solution was filtered through a 0.45-*μ*m syringe filter, and the filtrate was used as the test solution. Ten microliters of the test solution was injected into an HPLC system (Waters 2695 Alliance HPLC system; detector: Waters 996 PDA; column: Fortis C18 5 *μ*m 250 × 4.6 mm). The analysis involved an isocratic elution of acetonitrile and 0.1% formic acid (70/30, v/v) over 40 min at a flow rate of 1 ml/min.

### 2.4. Animal Experiments

Six-week-old C57BL/6 female mice were purchased from Orient Bio (Orient Bio, Seongnam, South Korea). The mice were group-housed (*n* = case) and allowed to acclimatize (object) for 1 week before treatment with VCD. Mice were randomly divided into five experimental groups (*n* = 8/group): negative control mice received an intraperitoneal (i.p.) injection of sesame oil daily for 15 days. VCD + phosphate-buffered saline (PBS) group, VCD + 17*β*-Estradiol (E2) group, and VCD + SF-treated groups (SF-treated mice were divided into three groups according to concentration of SF: 25, 100, and 400 mg/kg): mice received an i.p. injection daily of VCD (160 mg/kg) for 15 days. PBS was used as a vehicle control for SF. After dosing with VCD, PBS and each treatment were orally administered thrice a week during 6 weeks. Two days after the last dose (day 65), mice were sacrificed by CO_2_ asphyxiation, and uterus and serum samples were collected for analyses. No mice died during the experiment. All the experiments and animal handling procedures in this study were approved by the Animal Experimental Ethics Committee of the Kyung Hee University [KHUASP (SE)-16-037].

### 2.5. Body Weight and Tail Skin Temperature Analysis

The body weight and tail skin temperature of the mice were measured once a week for 9 weeks. The body weight was measured at the end of the experiment on an analytical balance for comparison between groups. The tail skin temperature was monitored during the sleep cycle using an infrared thermometer (11092060722, TPI 383, Summit, Korea). Three measurements were obtained 10 min apart, and the average value was used as a single data point.

### 2.6. Osteocalcin Analysis

Fasting serum samples were collected and stored at −80°C before analysis. The osteocalcin levels in the serum samples were determined using a commercial enzyme immunoassay kit (Takara Bio Inc., Seoul, Korea) according to the manufacturer's instructions. A 96-well microtiter plate was coated with an anti-mouse OC monoclonal antibody. The standard and sample were then added and incubated for 1 hat room temperature. A second ary peroxidase-labeled anti-Gla-OC monoclonal antibody was then added and incubated for 1 h. Finally, the plates were treated with the substrate solution (TMBZ) for 30 min, and the reaction was stopped with the addition of the TMBZ stop solution (50 *μ*L per well). Optical density of the samples was measured at 450 nm using a microplate reader (SoftMax PRO, version 3.1. software, CA, USA).

### 2.7. Serum Biochemistry Analysis

For serum biochemical analysis, the clotted blood samples were centrifuged for 10 min at 1,500*g* to obtain serum samples, which were analyzed using an automated analyzer (7080, Hitachi Ltd., Tokyo, Japan). The serum biochemistry parameters, including total cholesterol, triglyceride, low-density lipoprotein (LDL), and high-density lipoprotein (HDL), were analyzed.

### 2.8. Statistical Analysis

Statistical analysis of the data was conducted using Prism 5 software (GraphPad Software Inc., San Diego, CA, USA). All the values are presented as mean ± standard error of the mean (SEM). Statistical significance was determined by one-way ANOVA, followed by Tukey's test using randomly selected samples. A *p* value of <0.05 was considered statistically significant.

## 3. Results

### 3.1. Identification of SF

SF was characterized using Gomisin N by HPLC analysis (Figure 1(c)). By considering the amount of Gomisin N in SF, a calibration curve was constructed with six concentration levels (10.417, 15.625, 31.25, 62.5, 125, and 250 *μ*g/ml). Linearity was evaluated using the correlation coefficient (*R*^2^) of the calibration curve. Gomisin N was identified at a retention time of 25.085 min and its UV spectrum is shown in Figure 1(b). According to quantitative analysis, the yield of SF was 902.833 *μ*g/g.

### 3.2. SF Influences Menopause-Related Changes in Lipid Profile

**Figure 2:**
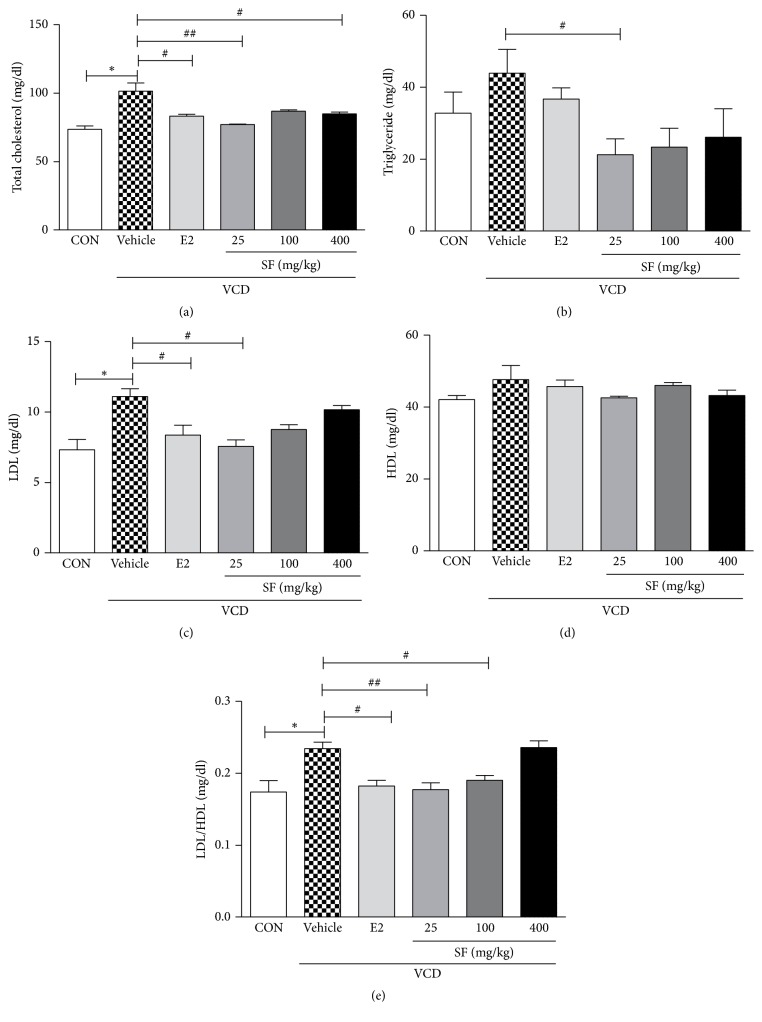
Effects of SF on changes in lipid profile. On day 65, serum was collected from all groups. (a) Total cholesterol, (b) triglyceride, (c) LDL, (d) HDL, and (e) LDL/HDL. Data are presented as mean ± SEM. *p* values are obtained using one-way analysis of variance (one-way ANOVA) by comparing the treatment group with VCD + Vehicle group. ^*∗*^*p* < 0.05 compared with the CON group; ^##^*p* < 0.01 and ^#^*p* < 0.05 compared with the VCD + Vehicle group.

On day 65, we collected serum samples from all groups to investigate the lipid profile. Total cholesterol, LDL, and LDL/HDL levels were significantly higher in the VCD + Vehicle group than in the control mice (Figures 2(a), 2(c), and 2(e)). Triglyceride and HDL levels were higher, but not significantly, in the VCD + Vehicle group than in the control mice (Figures 2(b) and 2(d)). However, the SF-treated groups had significantly lower total cholesterol, triglyceride, LDL, and LDL/HDL levels than the VCD + Vehicle group did (Figures 2(a), 2(b), 2(c), and 2(e)). However, there was no significant difference in HDL levels between the groups (Figure 2(d)).

### 3.3. SF Is Associated with Menopause-Related Changes in Body Weight and Tail Skin Temperature

**Figure 3:**
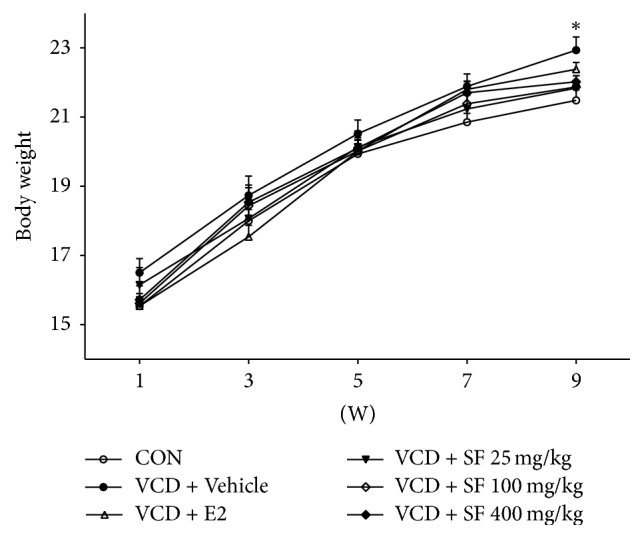
Time course of changes in body weight of mice. The body weight of mice was measured for 9 weeks. Data are presented as mean ± SEM. Differences between treatments were analyzed by one-way ANOVA with Tukey post hoc analysis. ^*∗*^*p* < 0.05 compared with the CON group.

**Figure 4:**
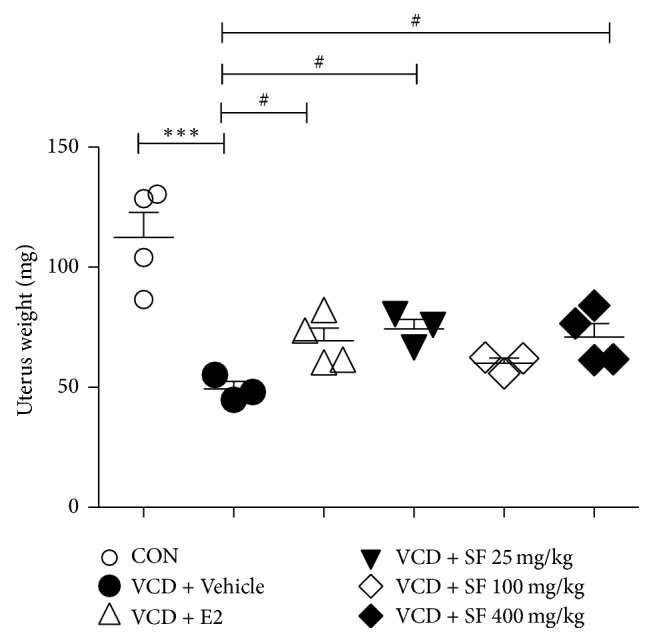
Effects of SF on changes in uterine weight. Uterine weight was measured at the end of the 9-week treatment period. Data are presented as mean ± SEM. *p* values are obtained using one-way analysis of variance (one-way ANOVA) by comparing the treatment group with VCD + Vehicle mice group. ^*∗∗∗*^*p* < 0.001 compared with the CON group; ^#^*p* < 0.05 compared with the VCD + Vehicle group.

We observed body weight and tail skin temperature for 9 weeks. After dosing (day 15), a gradual difference in body weight and tail skin temperature was observed in control mice and VCD-treated animals. From the eighth week, body weight of mice in the VCD group was significantly higher than that of control group mice (Figure 3). Mice in the SF-treated groups (25, 100, and 400 mg/kg) exhibited decreased body weight compared to that reported for VCD + Vehicle group mice. However, there was no significant difference (Figure 3). On day 65, we measured the uterine weight. The uterine weights of mice in the VCD + Vehicle group were lower than those of control group mice were. However, the uterine weights of mice in the SF-treated groups (25 mg/kg and 400 mg/kg) were higher than those of the VCD + Vehicle group mice were (Figure 4). The 100 mg/kg SF-treated group demonstrated a moderate increase compared to that reported for the VCD + Vehicle group. The tail skin temperatures were significantly higher in the VCD + Vehicle group than in those in the control group (Table 1). However, the temperatures in the VCD + E2 and SF-treated groups (25, 100, and 400 mg/kg) significantly decreased.

### 3.4. Inhibitory Effects of SF on Osteocalcin Production

**Figure 5:**
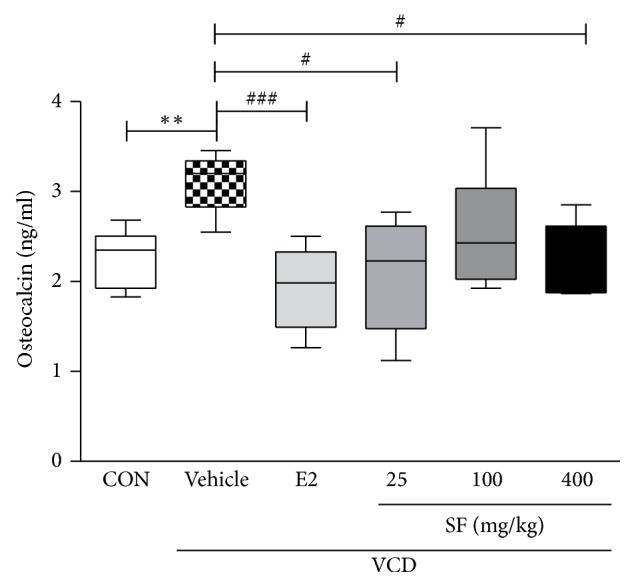
Effects of SF on changes in osteocalcin levels. On day 65, serum samples were collected from all groups. Data are presented as mean ± SEM. *p* values are obtained using one-way analysis of variance (one-way ANOVA) by comparing the treatment group with VCD + Vehicle mice. ^*∗∗*^*p* < 0.01 compared with the CON group; ^###^*p* < 0.001 and ^#^*p* < 0.05 compared with the VCD + Vehicle group.

Previous studies have demonstrated that osteoporosis is associated with menopause. After menopause, osteocalcin levels increase owing to the lack of sex hormones such as estrogen and androgen. Furthermore, osteocalcin is considered a bone turnover marker [24, 25]. Therefore, we hypothesized that SF prevents the development of osteoporosis by reducing osteocalcin levels. Serum osteocalcin levels in the VCD-treated group were significantly higher than those in the control group were. However, osteocalcin levels in the SF treatment groups were lower than those in the VCD + Vehicle group were, particularly in the SF 25 mg/kg and SF 400 mg/kg groups (Figure 5).

## 4. Discussion

Menopause is recognized by the cessation of menstrual cyclicity for at least one year [26, 27]. There are many factors that contribute to menopause, including age, health, environment, and lifestyle. This is caused by the depletion of germ cells and loss of ovarian follicular activity [6, 28]. In the previous report, authors suggested that impairment of BRCA1-related DNA double strand break (DSB) repair is associated with accelerated loss of ovarian follicular reserve and accumulation in mice and humans [29]. However, the exact mechanisms that cause menopause are still unknown. Menopause could lead to many diseases such as cardiovascular diseases [30], osteoporosis [31], ovarian cancer [8], and Alzheimer's disease [5]. The ovaries produce the sex steroids estrogen and progestins. However, as oocytes cannot be generated after birth, menopause occurs owing to the depletion of primordial follicles or ovarian failure [32, 33]. A clinical study reported some differences in symptoms of menopause between transitional hormone loss (natural menopause) and surgery. Compared with naturally occurring menopause, surgery-induced menopause was characterized by a lower memory score in women. The results suggested that surgery-induced menopause may have negative effects on brain function [34, 35]. With age, most women develop various diseases owing to a decrease in hormonal levels induced by menopause [35]. Many studies have used ovariectomy (OVX) and chemical induction (VCD) to generate models for studying menopause. OVX, a surgical method for removing ovaries, ceases hormone secretion. This increases the risk of osteoporosis and decreases cognitive function in women [11, 35]. On the other hand, VCD-induced follicle depletion mimics human menopausal transition [1]. VCD directly targets prenatal follicles and causes the loss of ovarian follicles in mice [16, 36, 37]. Furthermore, VCD can alter 17 *β*-estradiol level and estrous cyclicity [37]. According to some previous studies [10, 14], VCD-induced ovarian failure mice model, rather than OVX-induced model, is similar to menopause in humans. Symptoms induced by VCD, such as estrous cyclicity and changes in LH, FSH, and estrogen levels and a decrease in uterine and ovarian weights, resemble naturally occurring menopausal symptoms in women. Furthermore, VCD became known as a potential risk of human exposure to environmental ovarian toxicants in women [33]. Therefore, we used the VCD-induced ovarian failure mice model to observe the expected menopausal changes in women.

In clinics, HRT is widely used for the treatment of menopause-inducing symptoms. This therapy can alleviate menopausal symptoms and protect women from diseases such as osteoporosis and cardiovascular diseases [38, 39]. According to a previous study, HRT reduced the risk of colorectal cancer and Alzheimer's disease [40]. However, as HRT is estrogen-based, it can induce many other diseases such as breast cancer [7, 41, 42], ovarian cancer [8], gallbladder disease [9], endometrial hyperplasia [43], coronary heart disease [44], and venous thrombosis [45]. To resolve these limitations, herbal medicine could be used as alternate therapeutic strategy in the treatment of the menopause-associated diseases [46].

In many Asian countries, SF (Magnoliaceae family) is widely used in traditional medicine for the treatment of menopausal symptoms. SF is known as wu-wei-zi in Chinese [47], Gomishi in Japanese, and Omicha in Korea [48, 49]. It is associated with five flavors: sweet, sour, bitter, astringent, and salty flavors. Each of these flavors has various effects on different organs. Sour and salty components are believed to have good effects on the liver and testicles, bitter and stringent ones are believed to have good effects on the heart and lungs, and sweet component is believed to have good effects on the stomach [22]. In addition, numerous clinical trials have reported the efficacy of SF in conditions such as cough, dyspnea, dysentery, insomnia, amnesia, tumor, CNS, and cardiotonic disorder [18, 50, 51]. In Korea, SF is used in various herbal prescriptions for the treatment of menopausal symptoms, including Pheongbojinsimdan, Gamiondamtang, and Insugsan [18]. SF contains 13 lignans [52], including schisandrin [53] and Gomisin [54]. Among the ingredients of SF, schisandrin B (sch B) is the most abundant dibenzocyclooctadiene lignan, which is clinically used to treat hepatitis [55], cardiovascular diseases [53], and scopolamine-induced dementia [56]. According to a previous study, sch B can promote Treg proliferation, which secretes anti-inflammatory cytokines [57]. Furthermore, increased Treg suppresses bone resorption and osteoclast differentiation in bone marrow. Some articles reported that osteoporosis in postmenopausal women could be prevented [58, 59]. We also hypothesized that the administration of SF to VCD-induced ovarian failure mice could alleviate menopausal symptoms, such as an increase in body weight, tail skin temperature, and osteocalcin levels. Our present data demonstrated that the body weight of VCD group mice significantly increased after 8 weeks compared to that reported for the control group mice. Hot flashes are the most frequent symptoms of peri- and postmenopause period resulting from changing levels of estrogen in the body [60]. Hot flashes occur due to malfunction of the thermoregulatory system, which regulates body temperature and maintains homeostatic heat levels [61]. The underlying mechanism remains unknown. However, it is hypothesized that the rapid decline of estrogen at the time of menopause causes inducing hot flashes by influencing the action of nonepinephirine and serotonin. Tail skin temperature was also significantly elevated compared to that reported for the control group. Moreover, we observed a significant increase in uterine weight in the SF treatment groups compared to that in the VCD control group. It is well known that postmenopausal women have a high risk of weight gain [62] and type 2 diabetes [33, 63]. Because VCD is used to cause ovarian failure, uterine weight is typically decreased after VCD administration that is due to loss of tropic effects of ovarian estrogen on uterus. The reduction in uterine weight resulted from the withdrawal of ovarian estrogen and its well-known tropic effects on this target tissue. We also found the reduction of uterine weight after VCD administration compared to the control. However, the uterine weight gains in the SF-treated groups were lower than those of the E2 group. Increased uterine weight is a typical adverse effect of E2 therapy, because it may cause uterine cell proliferation and possible tumor development. Further examination for the exact mechanisms of SF on uterine weight will be needed.

Osteoporosis, a disease with decreased bone strength and increase in the risk of a broken bone, generally develops after menopause in postmenopausal women [64, 65]. Some previous studies suggested that osteocalcin serves as a major factor in osteoblast differentiation and can be used as a potential predictor of diabetes in postmenopausal women [66, 67]. Osteocalcin levels in the VCD treatment group were higher than those in the control group. However, a significant reduction in osteocalcin levels was observed in the SF treatment groups (25 mg/kg and 400 mg/kg). Based on these data, SF was suggested to effectively relieve various menopause-inducing symptoms in women.

## 5. Conclusion

Our data suggest that SF can successfully ameliorate menopausal symptoms in VCD-induced ovarian failure mice. SF could be used as a potential candidate for ameliorating symptoms of menopause in women.

## Figures and Tables

**Table 1:** Time course of changes in tail skin temperature of mice.

Tail skin temperature	1 weeks	3 weeks	5 weeks	7 weeks	9 weeks
CON	22.64 ± 0.22	22.16 ± 0.08	22.42 ± 0.17	22.34 ± 0.44	23.00 ± 0.13
VCD + Vehicle	22.80 ± 0.08	23.23 ± 0.08^*∗∗*^	23.63 ± 0.16^*∗∗*^	23.42 ± 0.19^*∗∗*^	23.27 ± 0.17
VCD + E2	22.25 ± 0.10	22.98 ± 0.20	22.45 ± 0.15^###^	22.57 ± 0.27^#^	22.75 ± 0.41
VCD + SF 25 mg/kg	21.94 ± 0.25^#^	21.66 ± 0.20^^###^^	21.91 ± 0.19^###^	22.79 ± 0.36	22.36 ± 0.27^#^
VCD + SF 100 mg/kg	21.80 ± 0.20^##^	21.52 ± 0.28^###^	22.64 ± 0.18^###^	21.91 ± 0.27^###^	22.09 ± 0.27
VCD + SF 400 mg/kg	21.60 ± 0.37^##^	21.63 ± 0.31^###^	21.28 ± 0.24^###^	21.55 ± 0.16^###^	23.00 ± 0.35

The tail skin temperature was measured at nine weeks. Data represent mean ± SEM. ^*∗∗*^*p* < 0.01, compared with the CON group. ^#^*p* < 0.05, ^##^*p* < 0.01, and ^###^*p* < 0.001 compared with the VCD + Vehicle group. Group and treatment differences were analyzed via one-way ANOVA with Tukey post hoc analysis.
